# A Wearable In-Ear EEG Device for Emotion Monitoring

**DOI:** 10.3390/s19184014

**Published:** 2019-09-17

**Authors:** Chanavit Athavipach, Setha Pan-ngum, Pasin Israsena

**Affiliations:** 1 Department of Computer Engineering, Faculty of Engineering, Chulalongkorn University, Phayathai Road, Wang Mai, Pathumwan, Bangkok 10330, Thailand; 2 National Electronics and Computer Technology Center, 112 Thailand Science Park, Phahonyothin Road, Khlong Nueng, Khlong Luang, Pathumthani 12120, Thailand

**Keywords:** EEG, in-ear EEG, emotion classification, emotion monitoring, elderly caring, outpatient caring, machine learning

## Abstract

For future healthcare applications, which are increasingly moving towards out-of-hospital or home-based caring models, the ability to remotely and continuously monitor patients’ conditions effectively are imperative. Among others, emotional state is one of the conditions that could be of interest to doctors or caregivers. This paper discusses a preliminary study to develop a wearable device that is a low cost, single channel, dry contact, in-ear EEG suitable for non-intrusive monitoring. All aspects of the designs, engineering, and experimenting by applying machine learning for emotion classification, are covered. Based on the valence and arousal emotion model, the device is able to classify basic emotion with 71.07% accuracy (valence), 72.89% accuracy (arousal), and 53.72% (all four emotions). The results are comparable to those measured from the more conventional EEG headsets at T7 and T8 scalp positions. These results, together with its earphone-like wearability, suggest its potential usage especially for future healthcare applications, such as home-based or tele-monitoring systems as intended.

## 1. Background

As societies around the world increasingly face with the issue of aging population, how to take care of these elderly people effectively becomes an important challenge. This is true especially for the less-fortunate ones who live alone. In order to ensure their physical and mental well-being and provide emergency assistance, monitoring technology could potentially be part of the solution. Particularly, wearable devices or smart sensors could be employed for effective and practical monitoring. Apart of conventional physiological signals, such as heart rate or EKG, that can be monitored to analyze the wearer’s health conditions, emotional state is one of the factors which reflects mental states and can greatly impact decision-making [1]. Emotion monitoring could therefore also be used as another piece of information for elderly and remote-patient caring supporting systems.

Emotion itself is very complex [2]. There are different interpretations for the many kinds of emotions, making emotion recognition far from straight forward. For research purposes, several simplified models have been proposed that can be categorized into two approaches; defining basic emotions and using a dimensional model. The most widely used basic emotions are the six basic emotions (i.e., anger, disgust, fear, joy, sadness, and surprise) generally used in facial expression recognition [3]. For the second approach, the common dimensional model is characterized by two main dimensions (i.e., valence and arousal). The valence emotion ranges from negative to positive, whereas the arousal emotion ranges from calm to excited [4]. This model has been used in a number of studies, because it is easier to express an emotion in terms of valence and arousal rather than basic emotions that can be confused by emotion names [5].

For a long time, most emotion recognition studies have focused on using facial expressions and speech. For continuous monitoring purposes, these approaches may not be the most suitable, as they may suffer from practical issues, such as ambient light and noises. Especially for camera-based facial recognition, the privacy issue is also a concern. Alternatively, physiological signals, such as galvanic skin response (GSR), electrocardiogram (ECG), skin temperature (ST), and electroencephalogram (EEG), which occur continuously and are harder to conceal, have been considered. As emotions are thought to be related with activity in brain areas that direct our attention, motivate our behavior, and determine the significance of what is going on around us, EEG, which is the signal from voltage fluctuations in the brain that are generated continuously at the level of cellular membranes [6], has been especially of interest. 

Emotion classification by EEG has been shown to achieve high accuracy [1,7,8,9,10,11,12,13,14,15,16]. However, most of those works employed multiple channel EEG headsets. In reality, these conventional multiple channel EEG standard headsets are not suitable for continuous monitoring due to their size and setup difficultly. Ideally, the EEG recording device used for emotion monitoring should be small, take little time to setup, and be comfortable to wear.

For such requirements, an in-ear EEG which is an EEG recording device introduced by Looney et al. in 2012 [17] could be of interest. Generally, the potential benefits of using an EEG of the in-ear type include the fact that it does not obstruct the visual field. It is also positionally robust, as it is generally fixed inside the ear canals. It is unobtrusive, as it is similar to devices people commonly use, such as earphones, earbuds, and earplugs. It is unlikely to encounter sweat, and also user-friendly for setup and maintenance. Unlike scalp EEG devices, which may require some experienced assistants to help, in-ear EEG devices could be simply put into users’ ears. However, an in-ear EEG also has some drawbacks. An in-ear EEG has much fewer electrodes and covers a much smaller area than what the scalp EEG can. So, its application accuracy is expected to be less than that of the scalp EEG. 

Our work was aimed at building an in-ear EEG device and evaluating it in terms of signal quality compared to those measured via scalp EEG at comparable positions (i.e., T7 and T8 based on the international 10–20 system [18]). The international 10–20 system is an internationally recognized system for labelling scalp locations for EEG measurement. The T7 position is located above the left ear, while T8 is positioned above the right ear. The prospect of an in-ear EEG usage for emotion classification was also investigated by experiments.

The paper is organized into six sections. Related works are discussed in Section 2. Section 3 describes material selections and system design. Detailed experimental protocols are included in Section 4. Experimental results and analysis are presented in Section 4. Significant findings from the results are discussed in Section 5. Finally, the conclusions are presented in Section 6.

## 2. Related Work

### 2.1. Scalp-Based EEG Emotion Classification

Scalp-based emotion classification by multi-channel EEG has been an active field of research [1,7,8,9,10,11,12,13,14,15,16]. A review of some of those works can be found in [7]. The majority of the works have focused on signal processing techniques to improve accuracy. For example, Koelstra et al. [19] presented methods for single trial classification using both EEG and peripheral physiological signals. The power spectrum density (PSD) of EEG signals was used as the primary feature. A support vector machine (SVM) classifier was used to classify two levels of valence states and two levels of arousal states. For EEG analysis results, average and maximum classification rates of 55.7% and 67.0% were obtained for arousal and 58.8% and 76.0% for valence. Huang et al. [20] developed an asymmetry spatial pattern (ASP) technique to extract features for an EEG-based emotion recognition algorithm. The system employed k-nearest neighbor (K-NN), naive Bayes (NB), and support vector machine (SVM) methods for emotion classification. The average accuracy rates for valence and arousal were 66.05% and 82.46%, respectively. We note here that several studies [7,21,22,23] have targeted the PSD of EEG data as the input features and performed emotion classification by using SVM. Other machine learning techniques, such as naive Bayes, K-NN, LDA, and ANN, have been applied in other studies [9,24,25,26].

Other areas of focus for scalp-based EEG emotion classification include those in [15,27], which look to develop wearable headband solutions. However, for monitoring purposes, these designs may suffer in conditions such as a warm climate; it might be uncomfortable to wear headband for a long duration due to sweating. Moreover, the sweat could affect the electrode impedance, resulting in noisy signal and inaccurate monitoring

### 2.2. In-Ear EEG Development

Originally, an in-ear EEG, which is an EEG recording device introduced by Looney et al. in 2012 [17], was demonstrated to have wearable characteristics that could potentially fulfill monitoring requirements [28]. It is small and could be worn around the ears, and is similar to earplugs or hand-free devices. Since then, research works have focused on areas such as materials; system design, especially in terms of practicality; and the verification of signal quality [17,27,29,30,31]. For example, Goverdovsky et al. [30] suggested a new prototype called Ear-EEG that consists of a viscoelastic substrate memory foam earplug and conductive cloth electrodes to insure conformance with the ear canal surface for motion artifacts’ reduction. Kullkami et al. [27] designed a soft and foldable electrode that can capture the EEG from different outer complex surfaces of the ear and the mastoid using the epidermal electronics with fractal mesh layouts. Recent work by Kappel et al. [31] developed an in-ear EEG with a soft earpiece, which required customized molding to fit individual ears. The prototype showed good signal quality and the potential for long term EEG monitoring.

### 2.3. In-Ear EEG for Control

In the field of brain–computer interface, artifacts in EEG signals created through muscle activity, such as eye blinks or other facial expressions, have been studied as a means for controlling external devices. For in-ear implementations, major works in the area include; Matthies et al. [32] which reported an in-ear headset based on a hacked NeuroSky EEG sensor. The prototype utilizes eye winking and ear wiggling for explicit control of the function of a smartphone. Additionally, in 2017, Matthies et al. [33] placed multi electrodes onto a foam earplug to detect 25 facial expressions and head gestures with four different sensing technologies. Five gestures could be detected with accuracy above 90%, and 14 gestures with accuracy above 50%. The prototype was also shown to be robust under practical situations, such as walking.

### 2.4. In-Ear EEG for Medical and Healthcare Applications

Medical and healthcare applications have also been a major theme for in-ear EEG research, especially for monitoring purposes [34]. Sleep has been particularly of interest [35,36]. For example, Nguyen et al. [35] proposed a dual channel EEG in the form of an earplug that showed a stable sleep stage classification with an average of 95%+ accuracy. In terms of emotion monitoring, which is closely related to this work, previous work [17,37] showed that an in-ear EEG signal measured was similar to T7 and T8 channels on the 10–20 system [18]. Moreover, one of the previous works also showed that T7 and T8 provided some informative data for emotion classification [7]. These results suggest that an in-ear EEG has the potential to classify emotions, which our work was to investigate.

## 3. Materials and Methods

In this work, to achieve the goal of realizing an in-ear EEG, we looked to find answers to these questions:

(1) What type of in-ear EEG should be studied (physically, design-wise, and engineering-wise)?
(2) What kind of EEG signal quality would we be getting?
(3) How good it is specifically for emotion classification?

For (1) we reviewed previous works and built some prototypes to evaluate their suitability. Once we decided upon the solution, we then moved on to verify the quality of measured signals compared to standard measurements to answer (2). It is important to do this before the main experiment as the result should be relatively comparable before we could move on to emotion measurement. To achieve that, we used the mismatch negativity (MMN) to compare auditory ERP measured via our ear EEG with those measured with a conventional headband EEG at T7 and T8 positions. Finally, for emotion classification, we needed reference to benchmark our measured results, so the DEAP dataset was used to calculate the accuracy of emotion classification at T7 and T8. It results were then used as reference for comparing with our own in-ear EEG measurements. All of this is explained in more detail in the following sections.

### 3.1. In-Ear EEG Development

#### 3.1.1. Earpieces Selection

**Figure 1:**
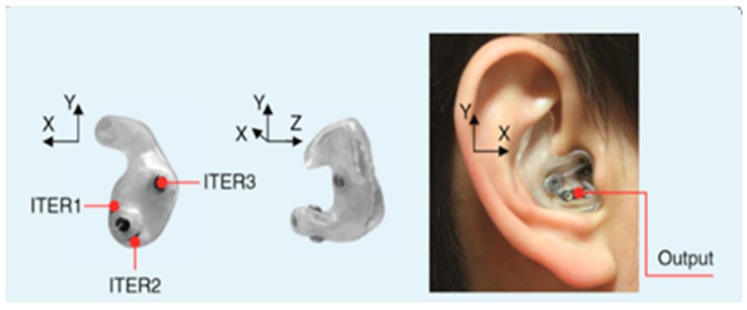
The first in-ear EEG prototype introduced by David Looney et al. in 2012 [17].

Recent research on in-ear EEG devices were studied [17,30,31,37]. There are currently 2 types of in-ear EEG devices; one is a personally customized earpiece, as illustrated in Figure 1, and the other is generic or non-customized. The first type is based on earmolds created from wax impressions, 3D scanning, CAD editing, 3D-printing, and a wiring process, respectively. This type of an in-ear device EEG is robust as it fits completely to the owners’ ear canal. However, it is relatively costly. Hence, this type of an in-ear EEG device was not considered in this study, as we would like a generic and low-cost device. 

**Figure 2:**
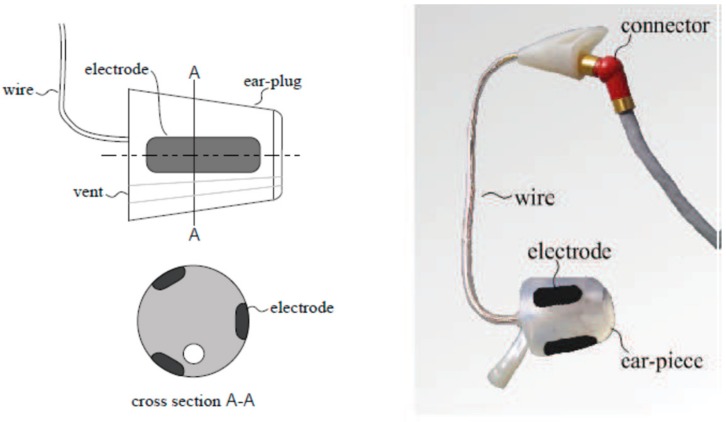
Generic in-ear EEG prototype [37]. The left side illustrates a drawing whereas the right side illustrates a model prototype.

The generic prototype is usually based on a cylinder-shaped material. The first generic in-ear EEG device was based on a cylinder of silicone, as illustrated in Figure 2 [37]. However, it has a flexibility disadvantage, as it is not guaranteed to fit into all ear canals [30]. The improved prototype used a cylinder-shaped memory foam instead of silicone. 

**Figure 3:**
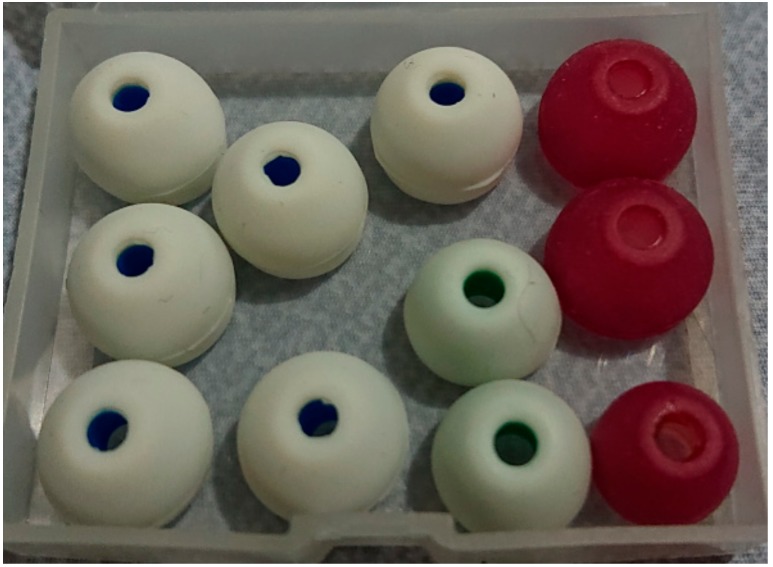
Different sizes of earphone rubbers.

Nevertheless, from our test, the in-ear EEG device built from memory foam ear-plugs could not fit into small ear canals. Furthermore, once fit in, it could also gradually slip out of the ear canal. Thus, in this study, the main body of the in-ear EEG device was changed to earphone rubbers, which were tested and found to have high flexibility. Additionally, they come in different sizes which could be properly selected to fit different ear canals, as shown in Figure 3.

#### 3.1.2. Electrode Selection

Three different materials were considered and tested for the in-ear EEG device electrodes, a half-sphere shaped silver, aluminum foil, and silver-adhesive fabric. The half-sphere shaped silver is probably one of the most widely-used materials for EEG electrodes. However, according to [30] the electrodes should be as similarly flexible as possible to the earpieces to achieve robust contact. Half-sphere silver is solid and not as flexible as the earphone rubbers. Therefore, the half-sphere silver was not selected. For aluminum foil, although it has low impedance and good flexibility, it could not be easily attached to electrical wires. This is because the aluminum foil is not adhesive to soldering. 

The silver-adhesive fabric, which was used with memory foam as in-ear EEG prototype [30], has flexibility similarly to memory foam and earphone rubber. It could also be glued and sewed to the wires without soldering. Therefore, the silver-adhesive fabric was considered suitable material for the electrodes for our in-ear EEG device. 

In this study, the size of the fabric was made slightly larger than in the previous study [30] for better contact. The fabric was glued to the ear rubbers, and the shield wires were then sewed to the fabrics. The number of the electrodes was also reduced to one channel per ear as the EEG signals among channels in the same in-ear from the previous studies were very similar [17]. The shield wire was slightly larger and heavier than a normal wire. However, it significantly reduced signal noise. Therefore, it was preferable to standard wire. 

**Figure 4:**
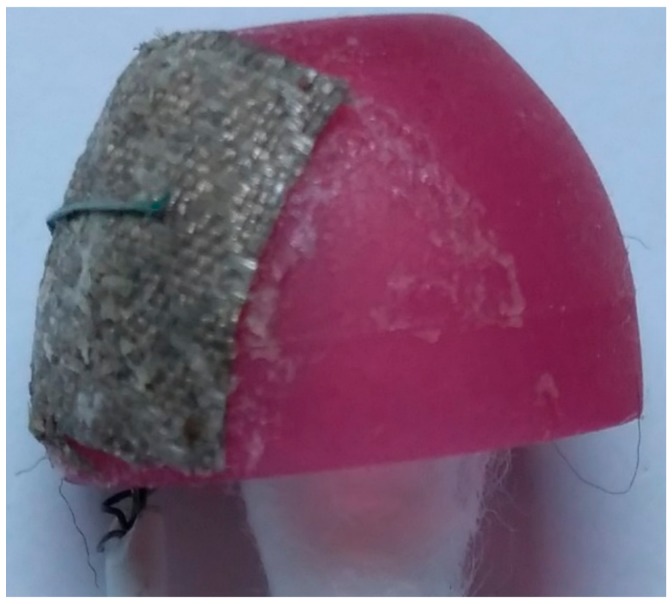
Single channel electrode used in the experiment using earphone rubber and silver-adhesive fabric electrode.

Our final prototype of in-ear EEG device is shown in Figure 4. The total material cost per piece is approximately 10 US Dollars. Our in-ear EEG device’s impedance was measured to be between 0.05 and 5.5 ohms which was comparable to that of OpenBCI electrodes: one of the commercial EEG electrodes [38].

### 3.2. In-Ear EEG Signal Verification

After the in-ear EEG devices were assembled, signal verification was performed. Mismatch negativity (MMN) is one of the widely-used methods for EEG verification [39,40]. It was used to verify in-ear EEG signals in the previous study [41]. Hence, it was also applied in our work. MMN is an experiment which observes the auditory event-related potential (ERP). ERP is a subject’s EEG signal response to an unexpected change of sensory stimulation. 

Our MMN experiment started by playing a short beep tone repeatedly until the subject was familiar to the tone. Unexpected mismatch tones were then inserted among the familiar tone. Unexpected mismatch tones could have a change of frequency (lower or higher), duration (unusually longer beep duration), intensity (unusually louder or lighter), or phase. The mismatch tone, if acknowledged, will provide an ERP response as a negative peak. The mismatch responses usually give a negative peak between 90 and 250 milliseconds after the beep [40]. The ERP latency may be varied according to personal musical experience [42].

The MMN experiment parameters in this study were set according to the previous study [40]. A combination of three pure tonal frequencies: 500, 1000, and 1500 Hz lasting for 75 milliseconds, were used as a standard tone, whereas two types of mismatch tones were applied. The first type was frequency mismatch containing 10% lower or higher pitch randomly applied to each frequency. The other type was a duration mismatch tone which lasted for 100 milliseconds, 25 milliseconds longer than the standard tone. The standard tone was beeped 15 times in order to make the subject familiar with the tone, before the mismatch tones were inserted. Mismatched tones arrived at the probability of 0.5, but no consecutive mismatch tones were allowed. 

The tones were played through an earphone. The in-ear EEG device was inserted to the right ear while the earphone was inserted to the left ear. The ground electrode was placed on the forehead and the reference electrode was placed on the right cheek, as suggested by [43]. An OpenBCI’s electrode was also placed at T8 as a comparison electrode. A Butterworth filter was used to notch 50 Hz powerline noise. It was also applied as a bandpass to filter the EEG signal between 2 and 30 Hz. The signal correlation between T8 and in-ear EEG was also calculated.

### 3.3. Emotion Model Emotion Stimuli

**Figure 5:**
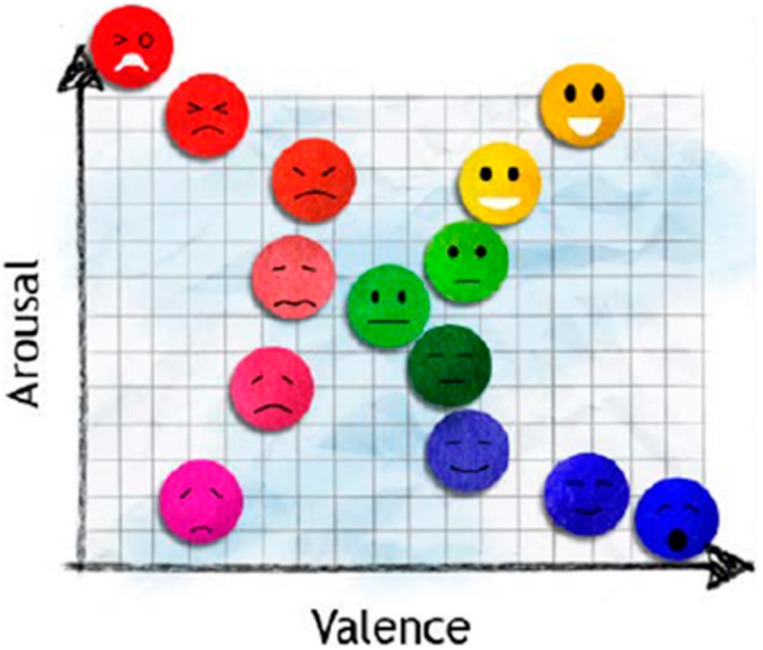
Valence and arousal model. Anger and fear have high valence and arousal. Happiness and excitement have high arousal and valence. Sadness and depression have low arousal and valence. Relaxation and pleasure have low arousal but high valence [4].

The valence and arousal emotion model [4], as in Figure 5, was used in this research, as it is a widely used simplified emotion model. Four emotions (happiness, calmness, sadness, and fear) will be classified according to the quadrants, respectively.

The International Affective Picture System (IAPS) [44], and the Geneva Affective Picture Database (GAPED) [45] were used as visual emotional stimuli. IAPS was the most widely used among previous research [1]. IAPS was developed at the Center for the Study of Emotion and Attention, University of Florida, by Lang, et al. [44]. IAPS pictures were standardized, and publicly available for use in emotional stimulation. The emotions elicited were based on two primary dimensions, which were valence and arousal. Valence ranged from unpleasant to pleasant, while arousal ranged from calm to excited. Every picture has valence and arousal rating from the scale 1 (lowest) to 9 (highest). However, IAPS contains fewer numbers of pictures stimulating low valence and low arousal than needed, so additional pictures from GAPED were used. 

The GAPED database was developed by Dan-Glauser, et al. at the University of Geneva [45]. It was intended to provide additional pictures to a limited number of IAPS for experimental researchers. GAPED provided a 730 picture database for emotion stimulation, which was also rated based on valence–arousal parameters as used in IAPS [44]. Moreover, four classical music pieces from auditory emotional research [46] were also applied as stimuli. The four musical pieces were also chosen based on the valence–arousal model, which corresponded to the IAPS and GAPED pictures.

### 3.4. Feasibility

Most previous studies on emotion classification used multiple EEG channels. The feasibility of emotion classification using a single-channel in-ear EEG should be evaluated first. The feasibility evaluation was conducted by performing an emotion classification experiment using secondary data from the Dataset for Emotion classification using Physiological and Audiovisual Signals (DEAP) [47]. DEAP data set is a publicly available dataset for Brain Computer Interface (BCI) based emotion study provided by Koelstra S., et al. [47]. 32 channel EEG data from 32 subjects was collected, while they watched music video clips that were chosen to elicit emotions. The emotions elicited were based on the valence–arousal model. Valence was associated with emotion positivity which ranged from unpleasant to happy/pleasant. Arousal was associated with excitement which ranged from calm to excited. The subjects rated the music video clips on valence–arousal scales. The DEAP dataset was hence labelled, and the classification accuracy on the data could be evaluated by the subjects’ rating. Out of 32 channels, only T7 and T8, which were stated to be close and correlate to the in-ear EEG were used for our emotion classification. Our emotion classification using DEAP dataset will be used for evaluating and comparing to the in-ear EEG emotion classification accuracy.

Support vector machine (SVM) which was widely used for emotion classification [1,7,10,16] was used as a classifier. SVM has good generalization and overfitting prevention properties. Therefore, it is considered suitable for this work. Six statistical parameters by Picard et al. [48] were used for signal feature extraction on a 3 s time-lapsed window. The Butterworth filter was used to notch 50 Hz noise, and filter EEG signals into five frequency bands; namely, delta, theta, alpha, beta, and gamma bands [6]. Ten-folded cross validation was applied to suppress biases [49].

### 3.5. Experiment Setup

This experiment was designed to collect EEG data using our in-ear EEG electrodes when subjects’ emotions were stimulated by pictures and sounds, described in Section 3.3. The results would be analyzed to assess the performance of in-ear EEG on emotion classification. 

Twelve male and one female subjects aged between 20 to 30 years with an average age of 24, were recruited for emotion classification experiments. Before the experiment started, the impedances of the in-ear EEG were re-measured as quality assurance. An in-ear EEG device was then inserted into either the right ear or left ear according to each subject’s preference, whereas earphones were inserted into the other ears. Earwax was cleaned by alcohol before the in-ear EEG insertion. 

Unless the subjects preferred to put the in-ear EEG on the left, it was put on the right ear as the left ear is shown to be better for listening to music [50]. The ground electrode was placed at forehead and the reference electrode was placed at either cheek inferior to the ear. A small amount of saline was used as electrolyte gel. Forty trials were recorded per subject. IAPS and GAPED pictures were randomly displayed to the subjects. The total number of pictures used for each emotion was as suggested by IAPS and GAPED datasheets. 

Each picture was displayed for 30 s. Subjects were recommended not to move during each picture viewing. Fifteen seconds of black screen was displayed after each picture in order to neutralize subjects’ emotions before the next picture was displayed. During the black screen subjects were free to mobilize. After eight pictures, subjects could have a small break and were free to move around before they were ready to continue. 

After the experiments were finished, the subjects were asked to evaluate their emotional response on each picture for emotion classification. This is because the emotional response to each picture may be different among subjects or different from the IAPS and GAPED datasets.

Statistical analyzes for any group comparison were performed using either *t*-tests or ANOVA, depending on the number of groups. A *p*-value of less than 0.05 was considered statistically significant. All statistics were performed using SPSS (IBM Corp., New York, USA)

## 4. Results

### 4.1. MMN Results

**Figure 6:**
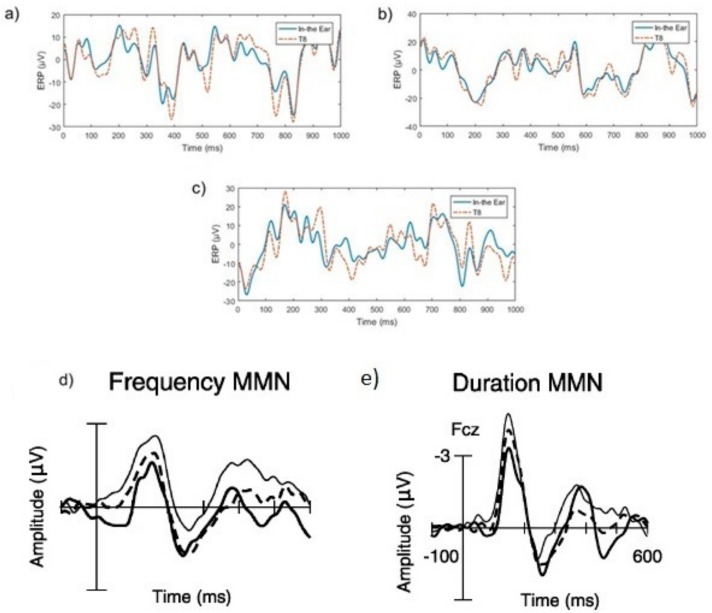
Examples of EEG after mismatch trials. (**a**) Example of frequency mismatch EEG event-related potential (ERP) response. (**b**) An example of duration mismatched EEG ERP response. (**c**) An example of an EEG ERP response after a standard beep. The blue and red lines in (**a**–**c**) show the in-ear and T8 EEG signal, respectively. (**d**,**e**) Duration and frequency mismatch responses from [40] for comparison. The dotted line in (**d**,**e**) show the ERP responses from similar traditional mismatch negativity (MMN) experiments to our work. The thin and thick lines in (**d**,**e**) show the MMN responses for specially designed experiments from [40].

Examples of frequency and duration mismatch responses compared to a standard tone are illustrated in Figure 6. In Figure 6a,b negative peaks between 200-400 ms which indicated mismatched ERP responses were found in both T8 and in-ear EEG signals. Different types of mismatched ERP signals, such as frequency and duration mismatched may vary in amplitudes, but general shapes of signals contain significant negative peaks around 200–400 ms [40]. These negative peaks of mismatch duration (Figure 6d) and frequency of mismatch (Figure 6e) from traditional MMN experiments, shown in the dotted line, from the previous study [40] are also shown in Figure 6 for comparison. The dotted lines in Figure 6d,e also show negative peaks between 200 and 400 ms. The examples of ERP responses to standard beeps are shown in Figure 6c. In contrast to the mismatch responses, the negative peaks are not present between 200 and 300ms. This conforms to the theory in [39]. 

Furthermore, the similarity between red and blue lines in all the plots in Figure 6a–c shows a high correlation between in-ear and T8 EEG signals. The correlation between T8 and in-ear EEG was approximately 0.8530 across all trials. These MMN results indicated that the signal measured by in-ear device was EEG, as its ERP response characteristics conformed to those of scalp EEGs. Additionally, in-ear EEG signal quality was similar to EEG measured at the nearby T8 scalp location.

**Figure 7:**
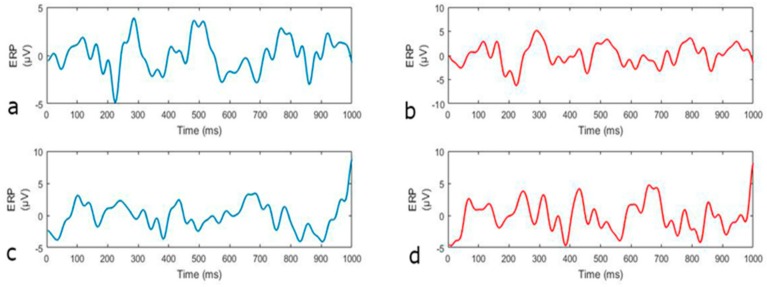
Average EEG of mismatch and standard trials. (**a**) Average in-ear EEG ERP responses from all mismatch trials. (**b**) Average T8 EEG from all mismatch trials. (**c**) Average in-ear EEG after standard beeps. (**d**) Average T8 EEG after standard beeps.

The average frequency mismatch response compared to the standard tone is displayed in Figure 7. The red and blue lines showed similar patterns (signs of slopes) between T8, and in-ear EEG. This result supports the findings of [16,36], which reports a high correlation between in-ear, and T7 and T8 EEG signals. It was noted that different amplitudes exist for the red and blue lines, because the signals shown were averaged across all trials, rather than raw data comparison (as shown in Figure 7a–c).

The MMN results show that in-ear EEG highly correlates with T7 and T8 EEG signals. Furthermore, similar signal response to the theory in [39] shows that in-ear EEG signal could be accurately used in a standard ERP test. Hence the validity of in-ear EEG signal was substantiated. 

### 4.2. DEAP Data Analysis

The emotion classification using T7 and T8 signals from DEAP dataset by SVM, as described in Section 2.4, was performed. Data from 32 subjects consisting of 40 trials per each subject were used for the classification. Ten-folded cross-validation was applied to suppress biases. In each classification, 36 trials were used as the training set and the other four were used for the test set. Ten different sets were trained and tested for each subject. 

The accuracy achieved was approximately 69.85 percent for valence classification and 78.7 percent for arousal classification. The overall accuracy for classifying four emotions was approximately 58.12 percent.

Furthermore, the analysis of emotion classification using the T7 or T8 channel was conducted and compared. The accuracies of emotion classification using T7 were approximately 71.30% for valence, 76.67% for arousal, and 57.56% for 4 emotions (valence and arousal combined); and the accuracy from emotion classification using T8 were approximately 70.93% for valence, 77.20% for arousal, and 57.34% for 4 emotions (valence and arousal combined) accordingly.

The *t*-test result from SPSS (IBM Corp., New York, USA) indicated that there was no statistically significant difference in classifying emotions between T7 and T8. The accuracy of T7 was approximately 57.56 ± 15.19 and T8 was 57.34 ± 16.40. The *p*-value was 0.955 on both tails, which was less than 0.955, indicating that there was no significance difference between classifying emotion using T7 and T8.

The results show that T7 and T8 data could be used as a single channel for valence, arousal, and the simple emotion classification, as the classification accuracy is comparable to the multichannel classification model in [7].

### 4.3. In-Ear EEG Emotion Classification

Only two out of thirteen subjects, subjects four and 10, decided to put an in-ear EEG on the left. The measurement of raw EEG data showed no statistically significant difference between EEG collected from left and right ear (*p*-value = 0.95). 

In-ear EEG signals were recorded while subjects were watching stimulating pictures during experiment, described in Section 3.5. The EEG signal was filtered using a 4th order Butterworth filter to notch out power line noise at 50 Hz. The signal was then separated into four frequency bands that were theta (4–8 Hz), alpha (8–12 Hz), beta (12–32 Hz), and gamma (30–48 Hz) by Butterworth bandpass filters. Six statistical parameters by Picard et al. [48] were used for signal feature extraction on a 3 s time-lapsed window. The SVM model described in Section 3.4 was used for classification. Ten-fold cross-validation was applied for classifying each subject’s data. All the signal processing and classification was performed offline using Matlab (The MathWorks, Inc., Natick, MA, USA)

Binary classification was done by SVM on valence (positive or negative) and arousal (high or low). The four emotion classification was performed using the valence and arousal classification results, mapped onto the simplified valence–arousal emotional model in Figure 5. For example, positive valence and high arousal was classified as happy. Hence the simplified emotions could be classified into four groups: positive valence/high arousal, positive valence/low arousal, negative valence/high arousal, or negative valence/low arousal. Classification accuracy was calculated by comparing SVM classifications with subjects’ own evaluations. The classification accuracy of in-ear EEG is shown in Table 1. 

The emotion classification accuracy based on the valence–arousal emotion model was approximately 73.01% for valence, 75.70% arousal, and 59.23% for all four emotions. Subjects four and 10 inserted the in-ear EEG on the left while the rest inserted it on the right. Subject 12 was female.

**Figure 8:**
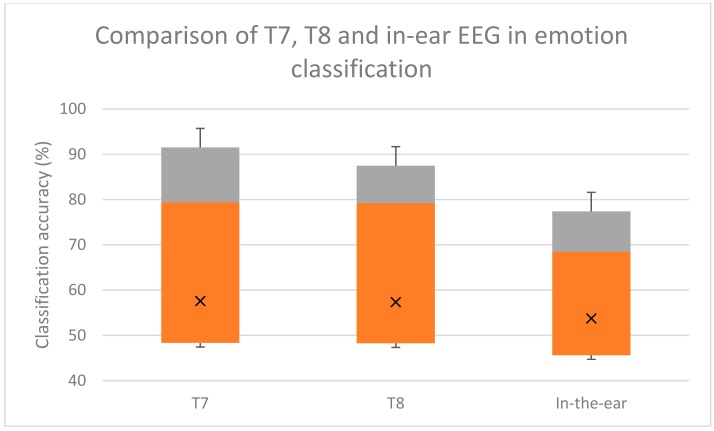
Box plot comparison among emotion classification using single channel T7, T8, and in-ear EEGs. Grey areas indicate proportions of classification accuracy above the median. Orange areas indicate proportions of classification accuracy below the median. X indicates the mean accuracy.

**Figure 9:**
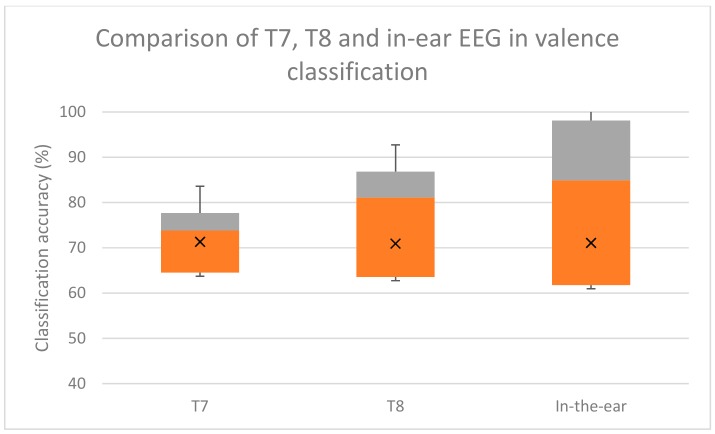
Box plot comparison among valence classifications using single channel T7, T8, and in-ear EEGs. Grey areas indicate proportions of classification accuracy above the median. Orange areas indicate proportion of classification accuracy below the median. X indicates the mean accuracy.

**Figure 10:**
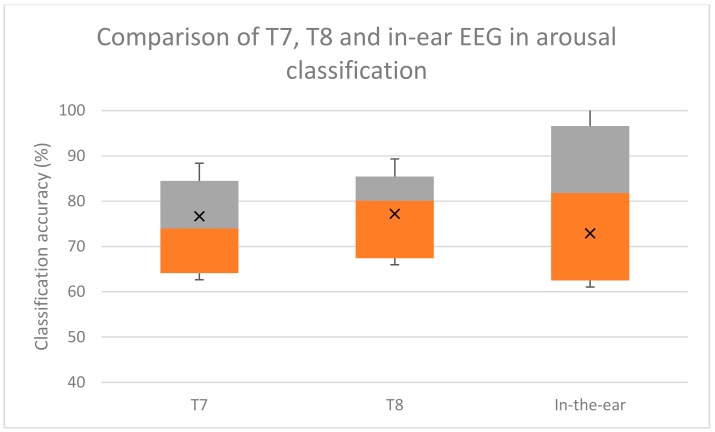
Box plot comparison among arousal classification using single channel T7, T8, and in-ear EEG. Grey areas indicate proportions of classification accuracy above the median. Orange areas indicate proportions of classification accuracy below the medians. X indicates the mean accuracy.

The accuracy of emotion classification using the in-ear EEG from our experiment, and the T7 and T8 EEG signals from the DEAP dataset were comparable. According to multiple comparison using Bonferroni test, there was no statistical significance difference between emotion classification using T7, T8, or in-ear EEG. The two-tailed *p*-values were 0.449 and 0.456, which was over the 0.05 threshold, indicating no significant classifying emotion using in-ear and T7/T8. The box-plot of the classification results are shown in Figure 8, Figure 9 and Figure 10.

Overall four emotion classification accuracies were approximately 53.72% for in-ear EEG and 58.12% for T7 T8 EEG. Valence classification accuracies were 71.07% and 69.85% for in-ear and T7 T8 EEG, respectively. Arousal classification accuracies were 72.89% and 78.7% for in-ear and T7 T8 EEG, respectively. These comparable accuracies indicate that in-ear EEG has potential for emotion classification as T7 and T8 electrodes do. 

## 5. Discussion

From the MMN results, in-ear EEG signal was verified to be highly correlated to the nearby T7 and T8 scalp EEG signals (correlation between T8 and in-ear EEG was approximately 0.853). This was expected as the 10–20 system scalp positions of T7 and T8 are just above left and right ears, respectively. They are in close proximity to ear canals. The results also correspond to the finding in previous work [17,37]. 

DEAP data analysis results show that using single electrode at T7 or T8 could achieve valence and arousal classification accuracies above 70 percent. This is comparable to classification accuracies obtained from using multiple EEG electrodes [7]. The results suggest that T7 and T8 could achieve a satisfactory emotion classification level. 

The results from 4.3 show that emotion classification accuracy from in-ear EEG was comparable to that of T7 and T8 (71.07% and 69.85% for valence, and 72.89% and 78.7% for arousal). The four emotion classification and arousal accuracies of in-ear EEG were slightly lower than those of T7 and T8 (53.72% and 58.12%). The valence classification accuracy was almost equal. 

Furthermore, the differences in accuracies in emotion, valence and arousal classifications between the in-ear EEG, and T7 and T8, are not statistically significant (*p*-values = 0.74, 0.99, and 0.65, respectively). Hence, an in-ear EEG is considered comparable to T7 and T8 in emotion classification.

From the above findings, in-ear EEG was found to be highly correlated to T7 and T8. Their emotion classification results are also compatible. Hence, in-ear EEG could be considered as an alternative to scalp EEG in positions close to the ears. 

In terms of wearability, in-ear EEG could be set up within five minutes and could be put on by the users themselves. During experiments most subjects did not complain of being uncomfortable or being disturbed during usage. It is also unaffected by sweat, which makes it suitable for long term monitoring in a warm climate. 

The additional benefits of the in-ear EEG are also in its compatibility and familiarity to users. Earplugs, earphones, and wireless handsfree earpieces have been around for many years and people are used to them. Wearing an earpiece is considered normal, so an in-ear EEG could allow the user’s acceptance much easier than conventional scalp EEG headsets. Another benefit of using in-ear EEG is the signal obtained has less artifacts from electrode movement compared to conventional scalp EEG. Scalp EEG headsets are susceptible to artifacts from the user’s movement, because contacts between the scalp and electrodes could easily become loose. With an in-ear EEG that fits tightly in the ear canal, body movement causes significantly less artifacts caused by loose contact between electrode and skin [30].

Compared to conventional scalp versions, the in-ear EEG is only a single channel device, with a similar signal to T7 and T8 scalp position near the ears. That limits in-ear EEG usage. Some EEG applications are not viable, such as for attention monitoring to measure the EEG from the frontal lobe [51]. Though this has never been investigated, in-ear EEG is not expected to achieve good accuracy in attention monitoring. 

A higher number of EEG channels could achieve higher accuracy in emotion classification [7], so it is a valid point to consider adding channels to the in-ear EEG. This could be done by adding more electrodes to the same earbud or wearing two in-ear EEGs on both ears. The former approach was developed in [17] with the use of a custom made earmold which is similar to the one used in a hearing aid. However, earmolds are much more costly than the generic earbuds used in this work, so additional signals would be gained at much higher costs. Furthermore, due to limited space in an ear canal, two electrodes placed there would be close together, hence similar signals are expected to be measured. The latter approach of wearing two in-ear EEGs on both ears is an alternative. It is probable that emotion classification accuracy would improve. The trade-off here is practicality for long term usage. A user who wears in-ear EEG on both ears will not be able to hear well, since both ear canals are blocked. Earbud redesign is needed to provide a gap in the middle to let sound through the ear canal.

Despite its potential, the in-ear EEG monitoring device would need to be further developed to be more practical. An additional feature required is wireless connectivity, possibly via Bluetooth. This would make it more convenient to use without cumbersome wires. However, the challenge is in the integrated circuit design, which needs to be able to fit into an ear canal. This point was also raised in [31]. 

## 6. Conclusions

An in-ear EEG device was developed. Earphone rubber was used as the in-ear EEG device main body. Silver-adhesive fabric was used as an in-ear EEG electrode. The in-ear EEG signals were verified to be close to T7 and T8 on MMN ERP responses, with a correlation of approximately 0.8530. The emotion classification results were approximately 71.07% for valence, 72.89% for arousal, and 53.72% for four emotions, compared to those of the DEAP emotion classification results using T7 and T8, which were about 69.85 % for valence, and 78.7 % for arousal, while the accuracy for classifying four simplified emotions was about 58.12%. Classification accuracies between in-ear EEG, and T7 and T8 electrodes, are not statistically significant. These results together with its earphone-like wearability, suggest its potential for novel healthcare applications, such as home-based or tele-monitoring systems.

## Figures and Tables

**Table 1:** Emotion classification result from each subject.

Subject	Valence	Arousal	4 Emotions
1	75.00%	69.64%	55.36%
2	89.58%	58.33%	47.92%
3	56.82%	77.27%	43.18%
4	75.00%	85.71%	71.43%
5	75.00%	59.37%	46.87%
6	86.54%	88.46%	76.92%
7	61.76%	70.59%	45.59%
8	86.11%	86.11%	72.22%
9	69.44%	91.67%	66.67%
10	38.64%	43.18%	22.73%
11	57.50%	62.50%	37.50%
12	75.00%	77.27%	54.54%
13	77.50%	77.50%	57.50%
**Average**	71.07%	72.89%	54.89%

## Abbreviations

EEG	Electroencephalogram
MMN	Mismatch negativity
IAPS	International Affective Picture System
GAPED	Geneva Affective Picture Database
DEAP	Dataset for Emotion Analysis using Physiological Signals
SVM	Support Vector Machine
